# Why P3HT Outperforms More Polar Analogues in OECTs

**DOI:** 10.1021/acs.chemmater.5c00737

**Published:** 2025-09-08

**Authors:** Priscila Cavassin, Tania Cecilia Hidalgo Castillo, Raymundo Marcial-Hernandez, Peter Gilhooly-Finn, Julien Réhault, Sahika Inal, Christian B. Nielsen, Natalie Banerji

**Affiliations:** 1 Department of Chemistry, Biochemistry and Pharmaceutical Sciences, 27210 University of Bern, Freiestrasse 3, Bern 3012, Switzerland; 2 Biological and Environmental Science and Engineering Division, Organic Bioelectronics Laboratory, King Abdullah University of Science and Technology (KAUST), Thuwal 23955-6900, Saudi Arabia; 3 Department of Chemistry, 4617 Queen Mary University of London, Mile End Road, London E1 4NS, U.K.

## Abstract

Materials that can conduct both ionic and electronic charges are known as mixed conductors. They are relevant for applications ranging from bioelectronics to neuromorphic circuits. A widely used strategy to enhance ion uptake and mixed ionic–electronic conduction in conjugated polymers is the incorporation of polar side chains. The increase in polarity, however, drastically affects film morphology and electronic transport, and their complex interplay is not yet fully understood. In this work, we investigate a series of poly­(3-hexylthiophene) (P3HT) analogues with an increasing content of ethylene glycol side chains to evaluate their impact on mixed ionic–electronic transport. Despite the higher polarity, the more glycolated copolymers exhibit a significantly lower electrolyte uptake. They also show lower electronic charge carrier mobility, and their performance in organic electrochemical transistors is drastically reduced. These findings challenge the current design rules for polymeric mixed condutors, as they show that higher polarity and disorder do not necessarily favor ionic uptake and highlight the key role of polymer packing when considering new side chain designs.

## Graphical abstract

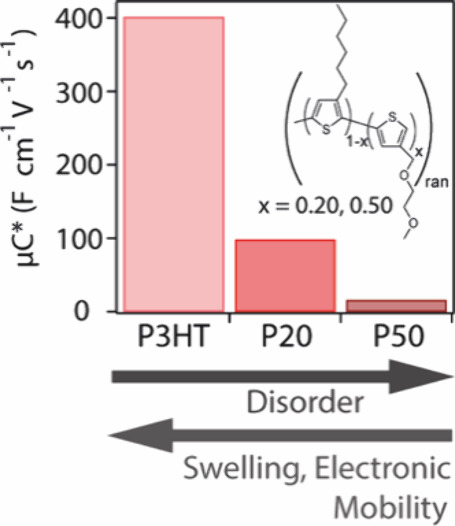

## Introduction

Conjugated polymers (CPs) are an important class of organic mixed ionic–electronic conductors (OMIECs). Recently, there has been a large interest in understanding the properties of these materials, not only because of their complex and still not well-understood mixed conductivity mechanisms but also due to the many applications that use CP-based OMIECs as active materials.^1,2^ The applications range from bioelectronics and electrochromics to supercapacitors and neuromorphics.^2,3^ Most of these applications rely on the process of electrochemically doping the CP film. In this type of doping, electronic charge carriers are injected into the film through a metal electrode, while counterions from an electrolyte interfacing the material are injected into the film, maintaining charge neutrality. For instance, the organic electrochemical transistor (OECT), which is an important platform for interfacing OMIECs with biological media, relies on the principle of electrochemical doping to facilitate efficient transduction from ionic to electronic signals.

Furthermore, because the OECT performance relies on the ability of a material to uptake ions (related to the volumetric capacitance, C*) and to efficiently transport electrons (the charge carrier mobility, μ), OECTs and their μC* figure-of-merit are typically used to benchmark CP-based OMIECs. There have been important advances in the understanding of how chemical structure modifications impact the performance of these materials.^4,5^ Notably, the strategy of substituting alkyl side chains on CP backbones for glycolated side chains has become the prevalent approach to improve OMIEC performance.^4–6^ Several studies have demonstrated with different polymer backbones that the incorporation of polar side chains improves aqueous electrolyte uptake, enabling polymers that were otherwise hydrophobic and could not be electrochemically doped to be well-performing OMIEC materials.^4,5,7^

In particular, the ability of the archetypical CP poly­(3-hexylthiophene) (P3HT) to be electrochemically doped has been well-documented, and several synthetic approaches to improve the efficiency of related polythiophenes have been demonstrated.^8–12^ Interestingly, P3HT is only poorly doped when interfaced with aqueous electrolytes containing small, hydrophilic anions such as chloride but can be efficiently doped when operated within electrolytes of larger, more hydrophobic anions such as PF_6_ or TFSI.^13,14^ Even though the synthetic efforts have improved the hydrophilicity of polythiophenes and their OECT performance in aqueous NaCl, these derivatives remain significantly more efficient in electrolytes with larger hydrophobic anions, such as PF_6_ and TFSI.^11,12^ While bioelectronic applications generally require operation in biocompatible electrolytes, such as NaCl or buffer solution, this is not critical for other applications such as electrochromic displays, neuromorphic devices, or supercapacitors. Therefore, optimizing the performance of readily available OMIECs such as P3HT in different aqueous electrolytes holds significant value.

In this work, we investigate a series of P3HT derivatives with increasing content of ethylene glycol polar side chains, which are randomly incorporated together with hexyl chains along the backbone, as shown in Figure 1 (see Table S1 for their molecular weight).^15^ We are interested in understanding the impact of the polar content on the aqueous KPF_6_-gated OECT performance, including electrolyte uptake, volumetric capacitance, and hole mobility. We find that the performance of the P3HT-based OECTs is excellent (μC* = 400 F cm^–^^1^ V^–^^1^ s^–^^1^), at least one order of magnitude higher than the more glycolated, polar copolymers. The μC* of P3HT is also better than or comparable to other glycolated P3HT analogues reported in the literature (doped in various aqueous electrolytes, Table S2).^10–12,16,17^ We explain the worse OECT performance of our more polar copolymers with the negative impact of disorder in the films. First, this reduces the electronic mobility, which has the most detrimental effect. Second, quite surprisingly, the disorder also reduces the water and ion uptake (leading to reduced electrochemical doping with both hydrophobic and hydrophilic anions). Third, the amorphous nature of the more polar films increases the oxidation onset potential. Our findings challenge the common belief that polar side chains and amorphous regions promote ionic transport and that water swelling is the main cause of reduced hole mobility in glycolated polythiophenes when doped in an aqueous KPF_6_ electrolyte.^11^ Instead, our results point to changes in film morphology, which are induced by increased hydrophilic content as a key factor affecting both swelling and electronic transport.

**Figure 1:**
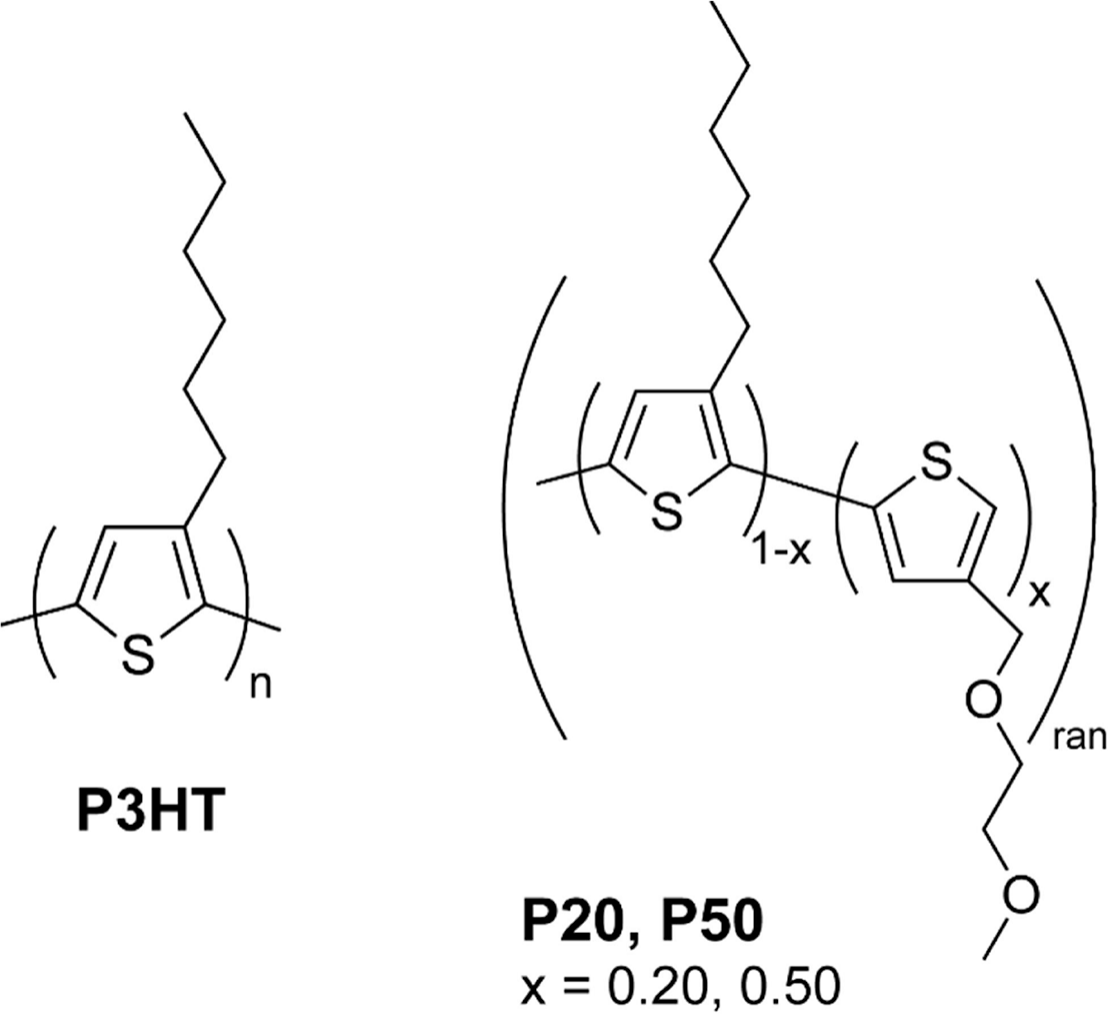
Structure of P3HT (left) and of random copolymers P20 and P50 (right).

## Results

### OECT Characterization

OECTs are commonly used as a platform to compare the mixed conduction performances of different OMIECs. The device transconductance (*g*_m_) is defined as the derivative of the drain current with respect to the gate voltage and is described by eq 1:

$$g_{m}=\mu C^{*}\frac{Wd}{L}(V_{\mathrm{th}}-V_{g})$$ 1

Here, μ is the electronic charge mobility, C* is the volumetric capacitance, $\frac{Wd}{L}$ is the device geometric factor (*W* = width, *L* = length, and *d* = film thickness), *V*_th_ is the threshold voltage, and *V*_g_ the gate voltage. The μC* product is an intrinsic characteristic of the OMIEC and electrolyte of choice and is typically used to benchmark the performance of these materials.^18^

We compare here the performance of the OECT of P3HT, P20, and P50 in an aqueous KPF_6_ electrolyte. The average molecular weights (*M*_n_) of P3HT, P20, and P50 are 55, 41, and 25 kg mol^–1^, respectively (Table S1). P20 and P50 films are more hydrophilic than P3HT films, as confirmed by contact angle measurements (Figure S1). Figure 2a shows the transfer curves and respective transconductances for the different materials. Output curves are shown in Figure S2, and the threshold voltages are shown in Figure S3b. We find that the device performance drastically worsens with an increasing polar side chain content. To quantify this difference, we measured 4–5 different channel dimensions for OECTs (as described in the Experimental Methods) of each material and plotted their maximum transconductance versus $\frac{Wd}{L}(V_{\mathrm{th}}-V_{g})$ (Figure S3). By linearly fitting each curve, we obtain μC* values for the three polymers. With μC* = 400 F cm^–^^1^ V^–^^1^ s^–^^1^, P3HT operated in KPF_6_ is among the best performing nonfused thiophene-based OMIECs reported to date. In Table S2, we summarize the OECT figures of merit of various P3HT derivatives (homopolymers and copolymers, measured in different aqueous electrolytes). This confirms that our P3HT-based OECTs outperform all glycolated homopolymers, including P3MEEET (poly­(3-[2-(2-methoxyethoxy)­ethoxy]­ethylthiophene-2,5-diyl)), that showed μC*= 332 F cm^–^^1^ V^–^^1^ s^–^^1^ when operated in KTFSI.^12^ TFSI^–^ is also a large anion that has been shown to dope P3HT even more efficiently than PF_6_^–^, yielding a higher source-drain current in OECTs.^13^

**Figure 2:**
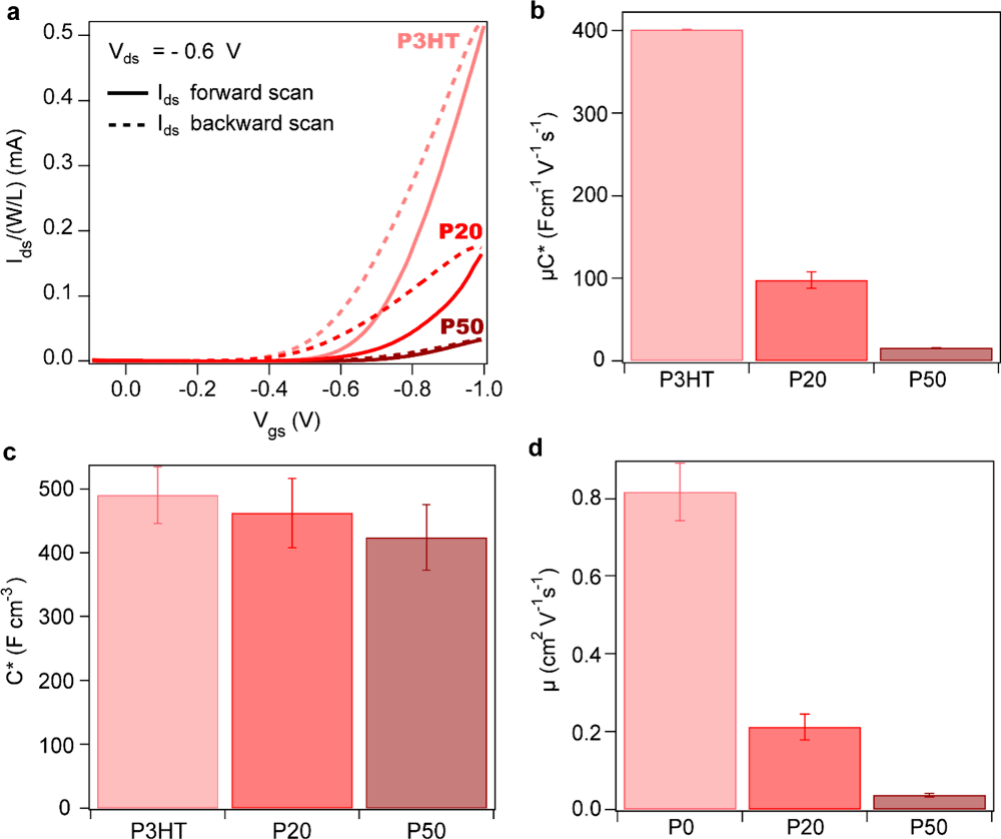
Overview of the OECT characteristics of P3HT (light red), P20 (red), and P50 (dark red). a) Forward (solid) and backward (dashed) transfer curves (scan rate of 3 mV/s) while applying *V*_ds_ = −0.6 V. The curves are normalized by the *W*/*L*. The forward scan was used for all the analysis. (b) Comparison of the μC* product, (c) of C* from chronoamperometry, and (d) of the inferred μ of P3HT, P20, and P50.

Only one report directly compares the OECT performance of P3HT and P3MEEMT.^11^ When operated in KCl, they found that P3MEEMT has a μC* of 49 F cm^–^^1^ V^–^^1^ s^–^^1^, five times higher than that of P3HT operated in the same conditions. In KPF_6_, P3MEEMT showed a higher μC* of 97 F cm^–^^1^ V^–^^1^ s^–^^1^, but the study did not include the corresponding μC* for P3HT operated in KPF_6_ and therefore did not compare the performance of both materials when using a more hydrophobic anion.^11^

Figure 2b compares the μC* products for P3HT, P20, and P50 operated in KPF_6_. We observe a 4-fold decrease in μC* from P3HT to P20 and a 5-fold decrease from P20 to P50. To better understand the origin of such a clear correlation, we use chronoamperometry to estimate the volumetric capacitance from the slope of the injected charge density as a function of voltage, as detailed in Figure S4a–c in the SI and previously described elsewhere.^19^ Contrary to the μC* value, the volumetric capacitances of P3HT, P20, and P50 are quite similar within experimental error. Comparing with P3HT, the P20 and P50 copolymers have 6 and 14% decreases in capacitance (Figure 2c). Thus, the volumetric capacitance does not improve by the presence of the polar side chains. Finally, by dividing the μC* product by C*, we estimate the electronic charge mobility within the assumptions of the Bernards-Malliaras model (Figure 2d).^20^ The hole mobility of P3HT is estimated to be 0.8 cm^2^ V^–^^1^ s^–^^1^, whereas for P20 and P50, there are dramatic decreases by 75 and 95% compared to the P3HT value, respectively.

This means that the OECT performance is drastically reduced in the polymers with higher polar content due to their inferior electronic transport. We hypothesize that this could be related to two main factors. First, a decrease in backbone planarity, degree of aggregation, and order observed in the polymers with increased polar content leads to worse electronic transport. The conductivity in OFETs has been investigated for this polymer series in a previous study, and they also observed a decrease in mobility.^15^ Another factor could be that the more polar films are prone to uptake more water, and excess water has been shown to be detrimental to electronic mobility.^21^ In the next sections, we further investigate these two hypotheses.

### Electrochemical Doping Processes

We measured the UV–vis absorbance of the dry films to gain some insight into the polymer chain ordering and aggregation of P3HT and the copolymers. Figure 3a shows a gradual blueshift in the absorption maximum, reaching a difference of approximately 13 nm between P3HT and P20 and of 59 nm between P3HT and P50, which is attributed to higher chain disorder, shorter conjugation length, and reduced intermolecular interactions with increasing polar content.^22^ P3HT has pronounced characteristic 0–0 and 0–1 vibronic bands centered around 606 and 556 nm, pointing to quite ordered chains with some H-type intermolecular interactions. The vibronic structure is more suppressed in P20, while P50 is essentially amorphous, as confirmed by the complete absence of vibronic bands.

**Figure 3:**
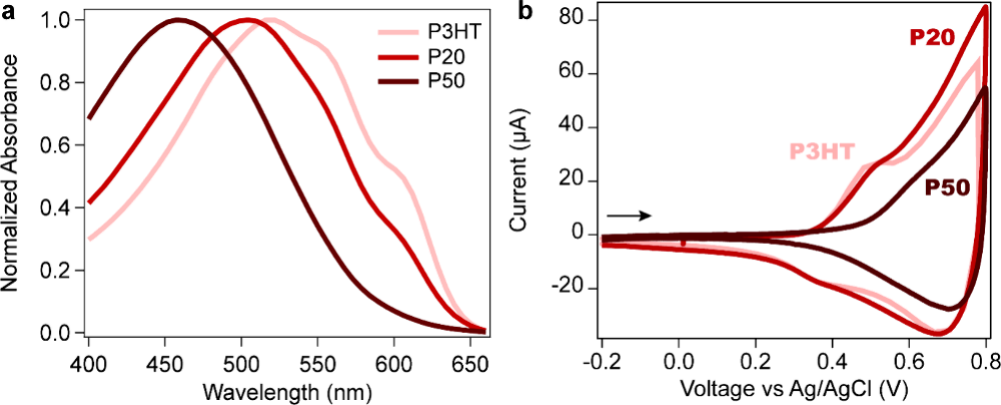
(a) Normalized absorbance of P3HT, P5, P20, and P50 thin films (≈30–40 nm dry thickness), from lighter to darker red. (b) Cyclic voltammetry of P3HT, P20, and P50 thin films in a 0.1 M KPF_6_ electrolyte with a scan rate of 50 mV/s. The arrow indicates the sweep direction.

A previous study led by Finn et al. used X-ray diffraction to investigate the film morphology of P3HT and a closely related series of random copolymers with 5, 10, 20, and 30% polar side chain contents.^15^ They demonstrated that P3HT crystallites orient edge-on relative to the substrate, exhibiting an in-plane π-stack scattering peak and multiple orders of strong lamellar scattering in an out-of-plane orientation. When the glycol content is increased, the polymers maintain their edge-on orientation. Qualitatively, they showed that the in-plane π-stack scattering intensity decreases with increased glycol content versus the scattering background, which indicates a lower degree of ordering.

The structural differences in the polymer films have a direct impact on their electrochemistry. Figure 3b shows the cyclic voltammetry curves of P3HT, P20, and P50 in an 0.1 M KPF_6_ aqueous electrolyte after three preconditioning 50 mV/S CV scans. The P3HT voltammogram is similar to those previously reported in the literature, which were measured in a TBAPF_6_ acetonitrile electrolyte.^23,24^ These studies ascribe the distinct peaks to the oxidation of different morphological domains (crystalline and amorphous), with Nightingale et al. suggesting a potential relation to the formation of singly charged polarons and doubly charged bipolarons. In P20, the oxidation onset takes place at similar voltages compared with P3HT, while P50, being completely amorphous, has a higher oxidation potential. The P50 voltammogram is very similar to that reported for the amorphous regiorandom P3HT.^24^ We noticed that after subsequent CV cycles and doping/dedoping steps, the P3HT and P50 voltammograms show significant differences (Figure S5), showing an oxidative current at a lower voltage, around 0.1 V, while P20 remains unchanged. Possible reasons for this new low current peak are changes in the film microstructure, oxidative degradation, or permanent doping.^25^ This is likely an indication that P20 is more stable than P3HT and P50.

Furthermore, by measuring the absorbance changes as we electrochemically dope our films under varying biases, we gain insight into the doping levels and the different charged species formed in the films. We measured the absorbance of P3HT, P20, and P50 films coated on conductive indium tin oxide (ITO) substrates in a 0.1 M KPF_6_ aqueous electrolyte using a spectroelectrochemistry setup. The steady-state absorbance changes observed after applying 30 s square voltage pulses from 0.1 to 0.8 V are shown in Figure 4a–c. At 0 V, the absorbance spectra of the wet films are similar to the ones of dry films in Figure 3a, pointing to weak disruption of the overall film structure due to passive swelling. For all films, increasing the doping (oxidation) voltage results in decay of the neutral band around 500 nm. In P3HT and P20, the decay is initially accompanied by the rise of a peak at 800 nm and a tail at higher energies (for voltages ≤ 0.4 V), which is associated with two polaronic transitions (P2 and P1) due to the formation of singly charged species within the film.^8,26^ As the doping voltage increases, the P2 polaron band further increases and then slightly decreases, while a new band at 1400 nm starts to emerge. We associate this with the formation of doubly charged species, likely bipolarons, based on a previous electron paramagnetic resonance (EPR) report.^23^ Because the polaronic and bipolaronic bands are overlapping and their distinction is not straightforward, we used multivariate curve resolution analysis (MCR) to decompose the measured spectra into spectral signatures and their respective concentrations. Following the protocol established in our previous study, we decompose the spectra into neutral disordered, ordered, polaron, and bipolaron species (Figure S6).^8^ The concentrations in Figure S6b–d show that in P50, unlike P3HT and P20, both polarons and bipolarons form simultaneously, similar to what we observed in our study on the doping of fully amorphous regiorandom P3HT.^8^

**Figure 4:**
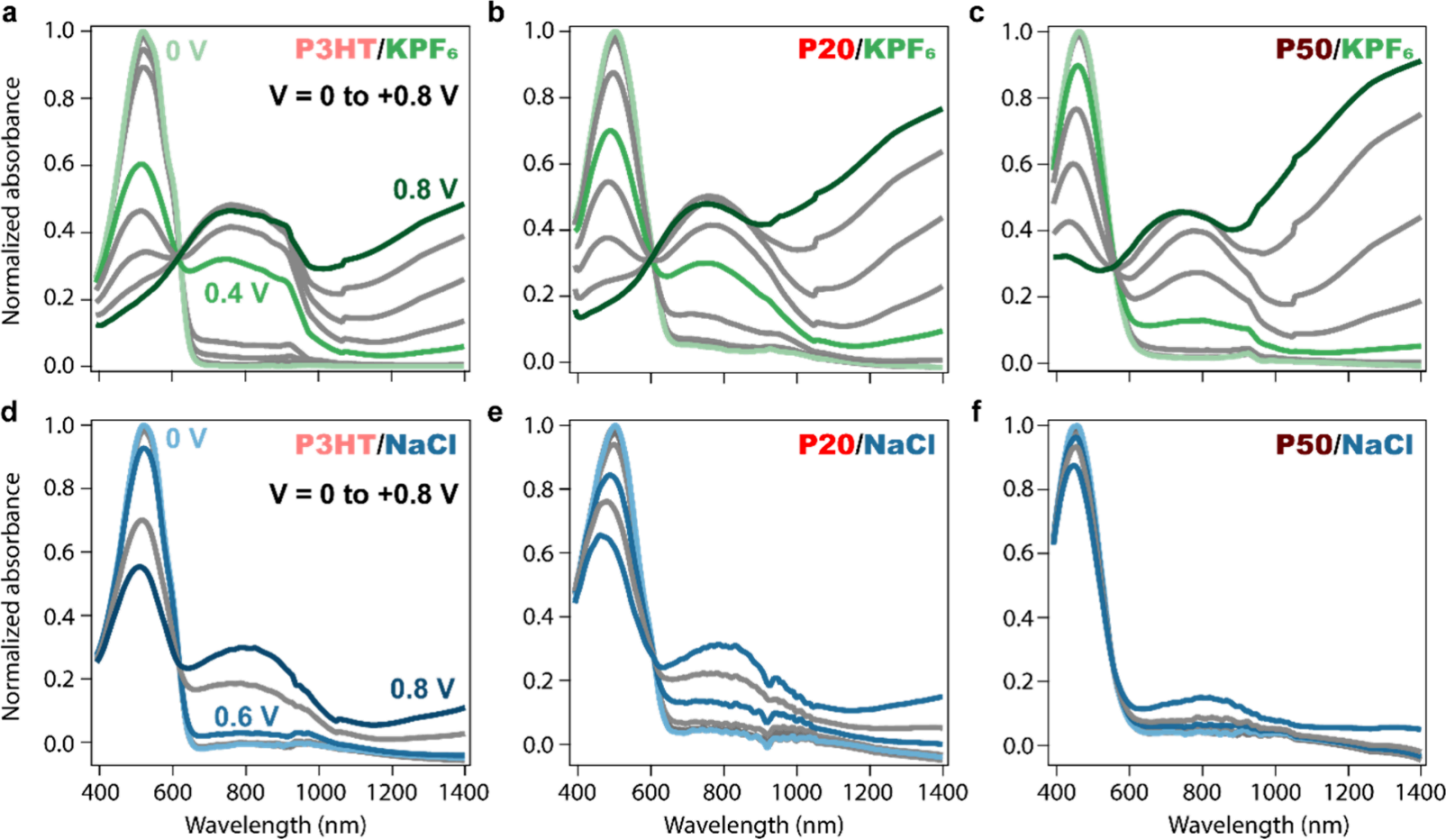
Normalized steady-state absorbance spectra of P3HT, P20, and P50 thin films doped in 0.1 M aqueous KPF_6_ (a, b, and c ≈ 100 nm dry thickness) and NaCl (d, e, and f ≈ 15 nm dry thickness) electrolytes. Each spectrum was acquired at the end of a 30 s square voltage pulse applied between 0.1 and 0.8 V, with steps of 0.1 V. The spectra were normalized by dividing them by the maximum absorbance at *V* = 0 V for each film. We note that the steep decay observed in the polaron band around 950 nm for the P3HT film is likely an artifact caused by our experimental setup.

Comparing the absorbance changes (Figure 4a–c) between the films doped in the KPF_6_ electrolyte gives us information about the extent of the doping for each material. We observe, for instance, that the neutral band decays by 80% for P3HT and P20 and by 70% for P50 at 0.8 V. The evolution of the neutral absorbance as a function of voltage is shown in Figure S6. The onset potential is similar for P3HT and P20, but 0.1 V higher for P50, which is also confirmed by the MCR concentrations shown in Figure S6. In the previous section, we determined the C* for the three polymers and showed that compared to P3HT, C* was slightly reduced in P20 and significantly smaller in P50, in agreement with the magnitude of the absorbance changes observed by spectroelectrochemistry. We can also observe that more bipolaronic species absorbing at 1400 nm are formed in the more polar (and more amorphous) films (Figure S7). In a previous study, we showed that bipolarons are preferentially formed in disordered polymer domains and that the polaronic transition at higher wavelengths and the bipolarons are blueshifted with disorder, which explains the higher absorbance around 1400 nm.^8,27^

The extensive literature available on the impact of the incorporation of polar side chains on OMIECs has shown that for a wide variety of materials, more polar polymers will present higher ionic/water uptake and therefore better OECT performance.^5,28^ In this study, however, we find that the volumetric capacitance in P50 is smaller than those found for P20 and even P3HT. We hypothesize that this might be the outcome of operating these measurements in KPF_6_, which is a more hydrophobic salt and therefore has a smaller hydration shell.^13,29^ Under these circumstances, including polar side chains would not be significantly beneficial. To test this hypothesis, we repeated the spectroelectrochemistry measurements using P3HT, P20, and P50 films, but now operated in aqueous NaCl (≈15 nm dry film thickness, because thicker films take very long to be doped in this electrolyte; see Figure S8). A study has shown that ions with larger crystallographic radii have smaller solvation radii. Chloride anions, therefore, are much more hydrated than PF_6_^–^ anions.^29^

The doping level in NaCl is always much lower than in KPF_6_ (and no operational OECTs could be obtained), but surprisingly, doping the films in NaCl does not change the trends observed in KPF_6_ and the P50 polymer with the most polar side chains again showing the lowest extent of doping (Figure 4d–f). Applying a potential of 0.8 V in NaCl results in a decay of 45% of the neutral peak in P3HT, 39% in P20, and only 13% in P50. The evolution of the neutral peak over the voltages is shown in Figure S7. The doping onset of P20 occurs 0.1 V before that of P3HT, an indication that, in NaCl, the doping is slightly more favorable after the addition of glycol chains. Similarly to what we observed in KPF_6_, P50 in NaCl has a much higher oxidation onset, at around 0.7 V. Moreover, there is no formation of bipolarons, indicating the lower level of doping that can be reached in NaCl, which is expected in P3HT due to the higher hydrophilicity of NaCl, but surprising for the polar copolymers.^13^ From these observations, we can conclude that even when doping the films with highly hydrated anions, the increased polar content does not translate into a higher ionic uptake and thus the doping level.

### Polymer Swelling and Mass Uptake

Because the most polar P50 film is less electrochemically doped than P3HT even in the aqueous NaCl electrolyte, we were interested in studying the total mass uptake of the films by using an electrochemical quartz microbalance with dissipation (E-QCMD). Several studies have demonstrated that polymers with polar glycol side chains typically uptake large amounts of water when compared to the corresponding nonpolar alkyl side chain polymers.^5,28^

In this work, we initially exposed the films to a KPF_6_ electrolyte. Once the system was stable (frequency changes smaller than 0.1 Hz per 5 min), we performed 4 CV cycles at a scan rate of 50 mV s^–1^ followed by voltage steps from 0.1 to 0.8 V versus the open circuit voltage (OCV, 0.08 V for P3HT, 0.01 V for P20, and 0.15 V for P50). Figures S9–S11 illustrate the changes in frequency, dissipation, and mass measured by E-QCMD. The mass is determined from the frequency data using the Sauerbrey model, during both the passive phase (prior to the application of any bias) and the active swelling phase. The dissipation is related to the rigidity/stiffness of the film. Changes in dissipation were minor, with a slight, irreversible decrease when voltage was applied, an indication of the rigidity of the films. During doping, the injection of PF_6_ anions resulted in an increase in the mass. When dedoping, a decrease in mass due to the ejection of anions was observed. Except for the mass retained during passive swelling, no mass accumulation was seen in the film in consecutive doping and dedoping cycles.

Figure 5 summarizes the passive and active swelling relative to the dry film while applying a voltage of 0.8 V versus the voltage of the OCV in the different films. P3HT swells about 20% when in contact with the electrolyte (Figure S12), and once doped, it reaches 38% relative to the dry film. P20 swells about 9% in contact with the electrolyte, and 20% once doped. Finally, we observed a swelling of less than 5% in the P50 films in contact with the electrolyte and of about 12% when doped. For both active and passive swelling, the trend is P3HT > P20 > P50. The number of injected ions calculated from the chronoamperometry current shows that, at *V* = 0.8 V vs OCV, the density of injected anions is approximately the same in P3HT, P20, and P50 (with a variation of <10% (Figure S4d), in agreement with the measured volumetric capacitance). This means that P20 and P50 uptake less water (and possibly cations) compared to P3HT, despite the higher polarity of their side chains.

**Figure 5:**
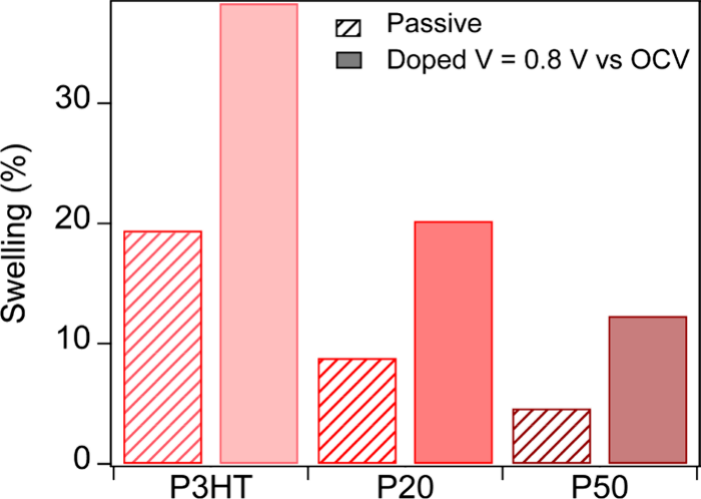
Comparison between the passive (before applying any voltage, slant lines) and active swelling (when applying 0.8 V, solid filling) in P3HT, P20, and P50.

## Discussion

Using different techniques such as OECT characterization, spectroelectrochemistry, and E-QCMD, we show that the more polar P3HT analogues have worse OECT performance in an aqueous KPF_6_ electrolyte, primarily due to lower electronic mobilities. This decrease is not due to a larger water uptake, which is typically detrimental to conductivity,^28^ but simply due to the increased film disorder. In contrast to its polar analogues, P3HT operated in aqueous KPF_6_ has an excellent OECT performance, comparable or surpassing the results demonstrated with several nonfused glycol-substituted polythiophenes operated in the same electrolyte.^10–12,16^ Surprisingly, we also observe that despite increasing the polar content in the films, they do not take up more ions, and P50 has a lower volumetric capacitance when compared to P3HT and P20. We also find that both P20 and P50 swell much less than P3HT when exposed to an electrolyte. After applying the voltage, their swelling remains significantly smaller than for apolar P3HT. Doping the films in a NaCl electrolyte (with more hydrated Cl^–^ anions) leads to even worse electrochemical doping in the P20 and P50 derivatives, as shown by the spectroelectrochemical data.

The drop in performance and swelling could be related to the higher number-average molecular weight (*M*_n_) of P3HT (55 kg mol^–1^) compared to P20 (41 kg mol^–1^) and P50 (25 kg mol^–1^). However, Tropp et al. published a systematic molecular weight comparison of glycolated polythiophenes (*M*_n_ varying between 13.9 and 32.5 kg mol^–1^) doped in hydrophobic and hydrophilic electrolytes and demonstrated that an intermediate molecular weight polymer (*M*_n_ of 22.4 kg mol^–1^) has the optimal μC*.^12^ Even though they observed that higher molecular weight resulted in more passive swelling, the relative differences we observed in our study (P3HT swells 120% more than P20) are much higher than the ones they reported (the highest *M*_n_ polymer swells 14% more than the lowest *M*_n_ polymer).

We initially expected that the more polar copolymers would uptake more ions and water compared to P3HT but found the opposite. A possible explanation is that the increased disorder in P20 and P50 counterbalances the benefits of the higher polar contents. Even though ionic transport is generally associated with the amorphous regions, with ion migration taking place through the electrolyte-swollen disordered parts,^1^ our results can be backed up by the literature.^10,30,31^ For instance, a similar result has been reported by Sivaraman et al.^30^ They studied a series of P3HT polymers with different regioregularities and electrochemically doped them with Et_4_NBF_4_ in an acetonitrile electrolyte. They also showed that the specific capacitance of P3HT increases with order and that the onset potential decreases. Another study compared three polythiophene homopolymers with differently linked (without, methyl and ethyl spacer) diethylene glycol side chains. They observed that the more ordered the polymer film is, the more electrolyte it absorbed (via E-QCMD) and the higher capacitance is achieved.^10^

Our results demonstrate that having more hydrophilic films is not enough to afford improved ionic capacitance and OECT performance, as detrimental structural changes may occur. The torsional disorder of the polymer backbone, which is enhanced here when substituting alkyl for glycol chains, could be at the origin of this observation. In P3HT, it is well-established that the alkyl side chains play a crucial role in the polymer assembly and planarity of the backbone.^32^ Moro et al. have shown that glycol analogues of poly­(2,5-bis­(3-alkylthiophene-2-yl)­thieno­[3,2-*b*]­thiophene) (pBTTT) tend to have more torsions or kinks in their backbone compared to their alkylated counterpart.^33^ Still, these analogues, namely poly­(2-(4,4′-bis­(2-methoxyethoxy)-5′-methyl-[2,2′-bithiophen]-5-yl)-5-methylthieno­[3,2-*b*]­thiophene) (pgBTTT) and poly­(2-(3,3′-bis­(2-(2-(2-methoxyethoxy)­ethoxy)­ethoxy)-[2,2′-bithiophen]-5-yl)­thieno­[3,2-*b*]­thiophene) (p­(g2T-TT), are among the best performing OMIECs in the field.^4^ We hypothesize that in P3HT, which, unlike pBTTT, has a nonfused thiophene backbone, the torsions induced by side chain substitution become even more relevant. This could result in a densely packed polymer structure, consequently reducing the available space for water and ion uptake even in the amorphous regions. Moreover, the disordered domains of P3HT films are doped at higher potential compared to the ordered regions, and thus, higher voltages are necessary to reach high doping levels.^8,34^ Therefore, while in fused thiophene backbone polymers, the incorporation of glycol side chain results in enhanced performance, this effect may not be replicated in polymers with nonfused thiophene backbones.

## Conclusions

We applied several techniques to investigate the role of increasing polar side chain content in P3HT analogues, including OECT characterization, spectroelectrochemistry, and E-QCMD measurements. When operated in a KPF_6_ electrolyte, P3HT was shown to be among the best performing nonfused polythiophene-based OMIEC to date, with a μC* of 400 F cm^–^^1^ V^–^^1^ s^–^^1^. This makes the readily available polymer a prime candidate for applications that do not require biocompatible electrolytes, such as electrochromic displays, neuromorphic devices, and supercapacitors. We show that the copolymers (P20 and P50) with higher polar content have worse OECT performance, primarily because of the increase in disorder, which is detrimental to electronic mobility. Furthermore, we also observe that the higher polar content does not translate into higher volumetric capacitance, with P50 having a smaller C* compared to P3HT. We attribute this to two main factors: (i) the more polar films uptake less water slightly less ions due to torsional chain disorder that might reduce the available space in the amorphous film regions, and (ii) higher voltages are necessary to reach high doping levels in the disordered film regions due to the higher oxidation potential.

Our work challenges the current material design rules for OMIECs, as we show that increasing polar content does not translate into better performance and that disorder does not necessarily favor ionic transport (excessive disorder even seems to be detrimental to ionic uptake). Our work also shows that depending on the electrolyte choice, P3HT derivatives with glycol substitutions are not more efficient than P3HT, an indication that generalizing the design rules to all backbones is not so simple. We stress that future material design should take into account the changes in order and polymer packing when comparing different synthetic side chains approaches.

## Experimental Methods

### Polymer Synthesis

P3HT and P20 were synthesized as previously described.^15^ For the synthesis of P50, a similar procedure was followed, as detailed here: In separate microwave vials, 2,5-dibromo-3-hexylthiophene (600 mg, 1.84 mmol) and 2,5-dibromo-3-(2-methoxyethoxymethyl)­thiophene (607 mg, 1.84 mmol) were dissolved in dry THF (5 mL) under N_2_ and then further degassed with N_2_ for 20 min. Then, to each vial, an isopropyl magnesium chloride lithium chloride complex (1.3 M in THF, 1.35 mL, 1.75 mmol) was added dropwise, and the two reaction mixtures were heated to 70 °C for 2 h. During this time, a separate flask was charged with Ni­(dppp)­Cl_2_ (10 mg, 0.04 mmol, 0.01 equiv) and placed under N_2_ after which dry THF (5 mL) was added. After the monomer solutions were allowed to cool to room temperature, they were added together in a new flask under N_2_ and the Ni­(dppp)­Cl_2_ suspension was subsequently added in one portion. The solution was heated to 70 °C overnight, then quenched with 1 mL of HCl (aq) (10% v/v), and stirred for 20 min. After most of the solvent was removed under reduced pressure, the crude polymer was precipitated into cold hexanes and filtered into a Soxhlet thimble. The crude polymer was purified by Soxhlet extraction, using methanol, acetone, hexanes, and chloroform. The chloroform fraction was precipitated by adding into cold hexanes and filtered, and then, the black solid polymer (246 mg, 43%) was dried under vacuum at 40 °C. The NMR spectrum of P50 is shown in Figure S13. Contact angle measurements were done using a tripod and a smartphone. The sample height and angle were adjusted so that the substrate was leveled when viewed through the phone camera.

### Sample Preparation

The polymers were dissolved in 1,2-dichlorobenzene (purchased from Sigma-Aldrich) with concentrations of 10 and 20 mg mL^–1^ for P3HT and P20 and 9 and 18 mL^–1^ for P50, to ensure similar film thicknesses after spin coating. Solutions were spin-coated at 1000 rpm for 60 s at room temperature on clean ITO-coated substrates (purchased from Ossila), OECT-patterned substrates (chromium/gold was evaporated into clean quartz substrates), or gold-coated QCM chips (purchased from Biolin Scientific). For OECT characterization and vis–NIR spectroscopy, the samples were placed in a homemade electrochemical cell with a 0.1 M NaCl or KPF_6_ aqueous electrolyte and a Ag/AgCl pellet electrode (purchased from Harvard Apparatus). The OECTs had different geometries, with the length varying from 0.2 to 2 mm and the width varying from 2 mm to 1 cm.

### OECT Characterization

OECTs were measured using a home-built setup controlled via custom-made LabVIEW software. The gate voltage was applied using a data acquisition system (USB-6211 NI), while the drain-source voltage was applied with a Keithley 2400 SMU. The gate and the drain-source current were measured with the NI system and Keithley, respectively. The voltage sign follows the transistor convention, where the source is grounded and a negative gate voltage induces p-type doping. The devices were gated with a Ag/AgCl electrode pellet. For all devices, we followed the same protocol. To increase reproducibility, the OECTs were initially precycled by applying four 30 s gate voltage pulses from +0.4 to −0.8 V. The device was dedoped for 30 s between each pulse. Then, we measured the transfer curves with *V*_D_ kept constant at −0.6 V and *V*_G_ ranging from +0.1 to −1.0 V and swept back with a speed of 3 mV/s. Next, we measured the output curves with *V*_G_ ranging from −0.4 to −0.9 V and steps of −0.1 V. *V*_D_ ranged from 0 to −0.8 V.

### Vis–NIR Spectroscopy

We used a home-built setup that allows biasing the film while simultaneously measuring the current and absorption spectra. The setup combined a halogen light source (HL-2000, Ocean Insight), a Flame UV–vis and a Flame NIR spectrometer (Ocean Insight) triggered by a digital delay/pulse generator (DG535, Stanford Research Systems), and customized LabVIEW code. A data acquisition card (USB-6008, National Instruments) was used to apply voltages between the pellet electrode and the film. The response current was converted to voltage using a low-noise current preamplifier (SR570, Stanford Research Systems) and measured by an acquisition card. The data were decomposed using the optimization method known as multivariate curve resolution (MCR), which is explained in detail in our previous publication.^35^

### E-QCMD

E-QCMD was measured with a Q-Sense analyzer (QE401, Biolin Scientific) using Cr/Au-coated quartz crystal sensors (area of 0.7854 cm^2^) and a Q-Sense electrochemical module (QEM 401) with a Ag/AgCl reference and the built-in platinum counter electrode. The bare sensors were initially measured in air and in a KPF_6_ 0.1 M aqueous electrolyte. This protocol was repeated for the coated sensors. For both the dry and wet measurements, enough time was given so that the frequency and dissipation were stabilized (Δ*f* < 0.1 Hz per 5 min). Once frequency was stable, the films were biased using a Biologic VMP-300 potentiostat. For each material, we first recorded the OCV and then performed three CV cycles at a scan rate of 50 mV/s followed by chronoamperometry steps ranging from −0.1 to −0.8 V vs OCV. After each step, the film was dedoped at 0 V vs OCV. The Sauerbrey model was used to convert the shifts into mass. Sauerbrey is considered an appropriate model because our Δ*d*/Δ*f* was smaller than 4 × 10^–7^.^36^ QSoft software was used to “stitch” the data to calculate the film mass for both dry and swollen. The swelling is the percentage change in volume relative to the dry film (*V*_dry_), as follows:

$$\mathrm{swelling}=\frac{V-V_{\mathrm{dry}}}{V_{\mathrm{dry}}}\times 100\%$$

## Supplementary Material



## Data Availability

The data that support the findings of this work are available as open access in the BORIS Repository of the University of Bern at https://boris-portal.unibe.ch/handle/20.500.12422/217214.

## Notes

Average molecular weight, contact angle measurements and ^1^H NMR spectra for all polymers, summary of OECT and material performance metrics of P3HT analogues, additional transistor characterization for all polymers, calculated injected charge and spectral decomposition for all polymers, P3HT film doping kinetics comparison in KPF_6_ and NaCl electrolytes, and E-QCMD data related to all polymers (PDF)
