# Supplementary material for: Why P3HT Outperforms More Polar Analogues in OECTs
Source: Chem Mater. 2025 Sep 8;37(18):6983–90. doi: 10.1021/acs.chemmater.5c00737 (PMC12461834; doi:10.1021/acs.chemmater.5c00737)
Supplement: Supplementary file 1 [file cm5c00737_si_001.pdf]

Supporting Information

Why P3HT outperforms more polar analogues in OECTs

Priscila Cavassin[1], Tania Cecilia Hidalgo Castillo[2], Raymundo Marcial-Hernandez[3], Peter Gilhooly-Finn[3], Julien Réhault[1], Sahika Inal[2], Christian B. Nielsen[3], Natalie Banerji[1]

[1] Department of Chemistry, Biochemistry and Pharmaceutical Sciences, University of Bern, Freiestrasse 3, Bern, 3012 Switzerland

[2] Biological and Environmental Science and Engineering Division, Organic Bioelectronics Laboratory, King Abdullah University of Science and Technology (KAUST), Thuwal, 23955–6900 Saudi Arabia

[3] Department of Chemistry, Queen Mary University of London, Mile End Road, London, E1 4NS UK

*natalie.banerji@unibe.ch

Table S1: Average molecular weight (M_w) and number-average molecular weights (M_n) in kg mol^{-1} and dispersity (Đ = M_w/M_n) of P3HT, P20 and P50.

	M_w/M_n (Đ)
P3HT	107/55 (1.94)
P20	58/41 (1.41)
P50	47/25 (1.88)

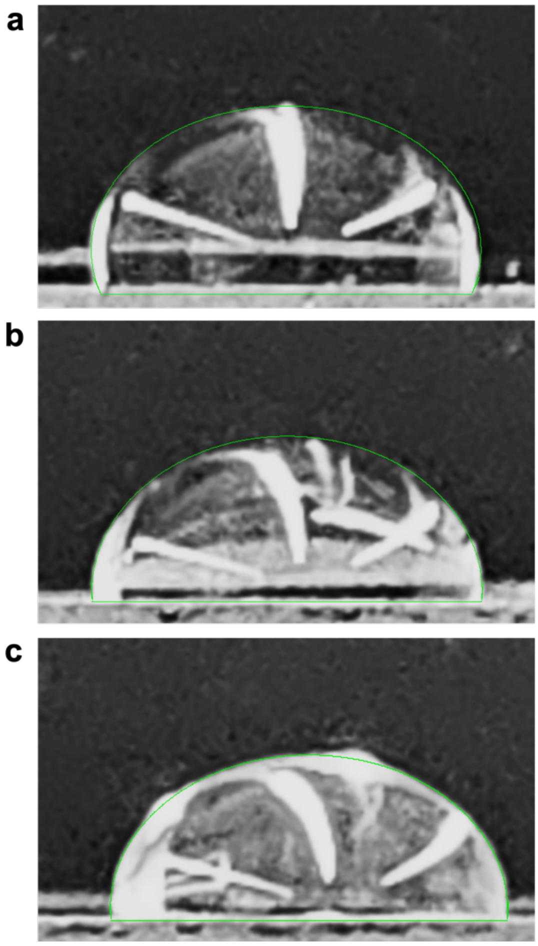

Figure S1: Contact angle measurements for a) P3HT (114°), b) P20 (95°) and c) P50 (96°).

Table S2: Summary of OECT and material performance metrics of P3HT-analogues (homopolymers and copolymers).

P3HT and analogues	μC* (F cm^{-1} V^{-1} s^{-1})[a]	C* (F cm³)[b]	μ (cm² V^{-1} s^{-1})[c]	Vth (V)	Electrolyte
P3HT (spin coated from chlorobenzene)[1]	10.4 ± 3.1	-	-	-	0.1 M KCl
P3HT (blade coated from carbon disulfide)[2]	93 ± 12	-	0.9 cm	−0.50[d]	0.1 M KPF_6
P3MEEET[3]	332 ± 37.7	156 ± 1	2.14 ± 0.153	-0.19[d]	0.02 M KTFSI
P3MEEET[3]	31.4 ± 5.8	219 ± 38	0.14 ± 0.009	-0.54[d]	0.1 M NaCl
P3MEEET[4]	11.5 ± 1.4	242 ± 17	0.05 ± 0.007	-0.57[d]	0.1 M NaCl
P3MEEMT[1]	96.7 ± 10.2	294	0.33 ± 0.034	-	0.1 M KPF_6
P3MEEMT[1]	49.1 ± 5.0	175	0.28 ± 0.029	-	0.1 M KCl
P3MEEMT[4]	9.8 ± 1.1	160 ± 12	0.06 ± 0.008	-0.66[d]	0.1 M NaCl
P3MEET[4]	0.04 ± 0.01	80 ± 9	0.0005 ± 0.0001	-0.18[d]	0.1 M NaCl
P3MAPPT[5]	41.3 ± 2.8	152.0 ± 21.1	0.27 ± 0.06	-0.27[d]	0.1 M KPF_6
	30.5 ± 2.2	81.0 ± 12.8	0.38 ± 0.09	-0.58[d]	0.1 M KCl
P3MAAPT[5]	33.2 ± 3.3	167.2 ± 7.7	0.20 ± 0.03	-0.35[d]	0.1 M KPF_6
	9.2 ± 2.8	25.4 ± 1.7	0.36 ± 0.13	-0.56[d]	0.1 M KCl
P3MPAAT[5]	13.3 ± 1.4	84.0 ± 5.9	0.16 ± 0.03	-0.47[d]	0.1 M KPF_6
	NA	1.3 ± 0.4	NA	NA	0.1 M KCl
p(g3T2)[6]	161	156 ± 1	0.90 ± 0.10	0.03	0.1 M NaCl
Copolymers					
PT2gT[7]	290 ± 80	290	1.00 ± 0.278	−0.540 ± 0.008	0.1 M KCl
p(g2T2-T)[8]	9 ± 2	8 ± 2	~10-4*	-0.20 ± 0.01	0.1 M NaCl
p(g3T2-T)[8]	135 ± 9	211 ± 18	0.16 ± 0.01	-0.18 ± 0.01	0.1 M NaCl
p(g4T2-T)[8]	54 ± 8	192 ± 10	0.06 ± 0.01	-0.19 ± 0.01	0.1 M NaCl
p(g2T2-g4T2)[6]	522	187 ± 8	1.72 ± 0.31	0.02	0.1 M NaCl
p(g1T2-g5T2)[6]	496	133 ± 3	2.61 ± 0.30	0.1	0.1 M NaCl
p(g0T2-g6T2)[6]	302	74 ± 4	2.95 ± 0.37	0.18	0.1 M NaCl

[a]extracted from the slope of the transfer curves. [b]extracted by electrochemical impedance spectroscopy (EIS). [c]obtained via dividing μC* by C*. [d]defined as the x-axis intercept of $\sqrt{I_{DS}}$ vs. V_{GS} plot, otherwise no method specified.

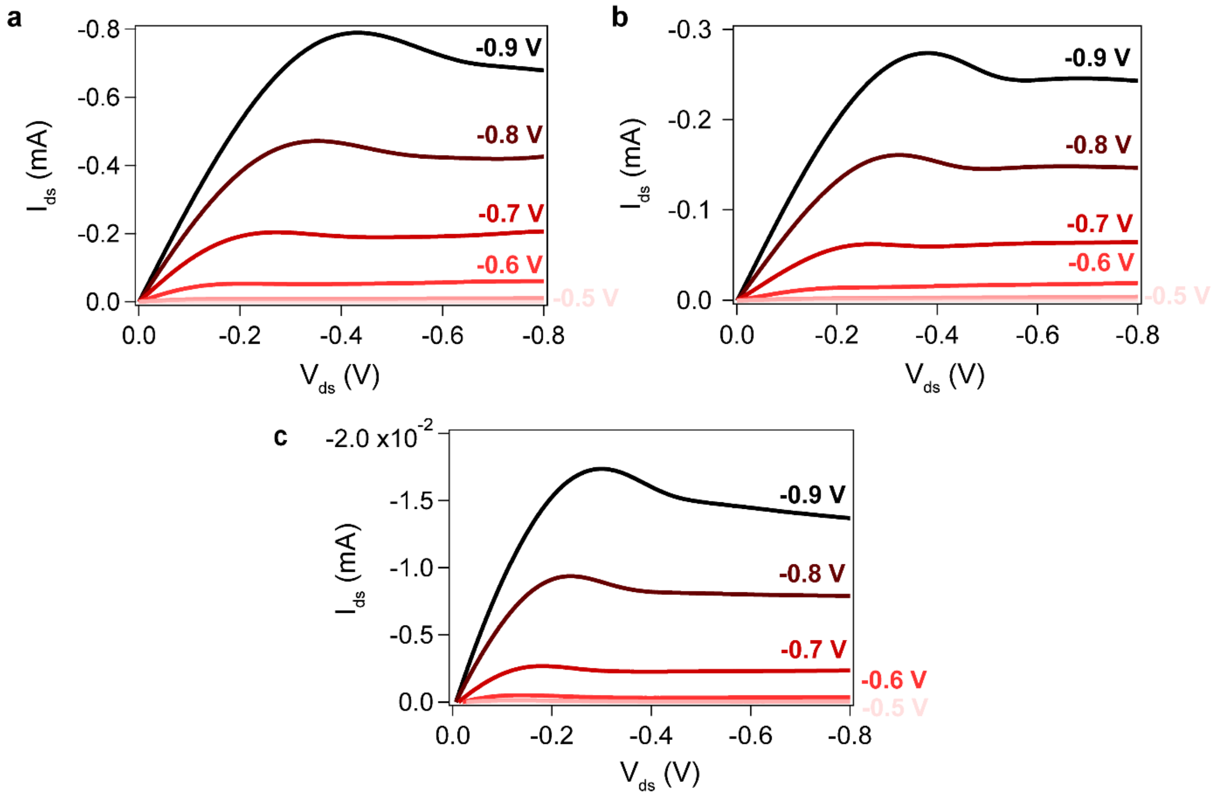

Figure S2: Output characterization for a) P3HT OECT with W/L = 1.5, b) P20 OECT with W/L = 1.5 and c) P3HT P50 with W/L = 0.9. The applied gate voltages for each curve are shown in the graph.

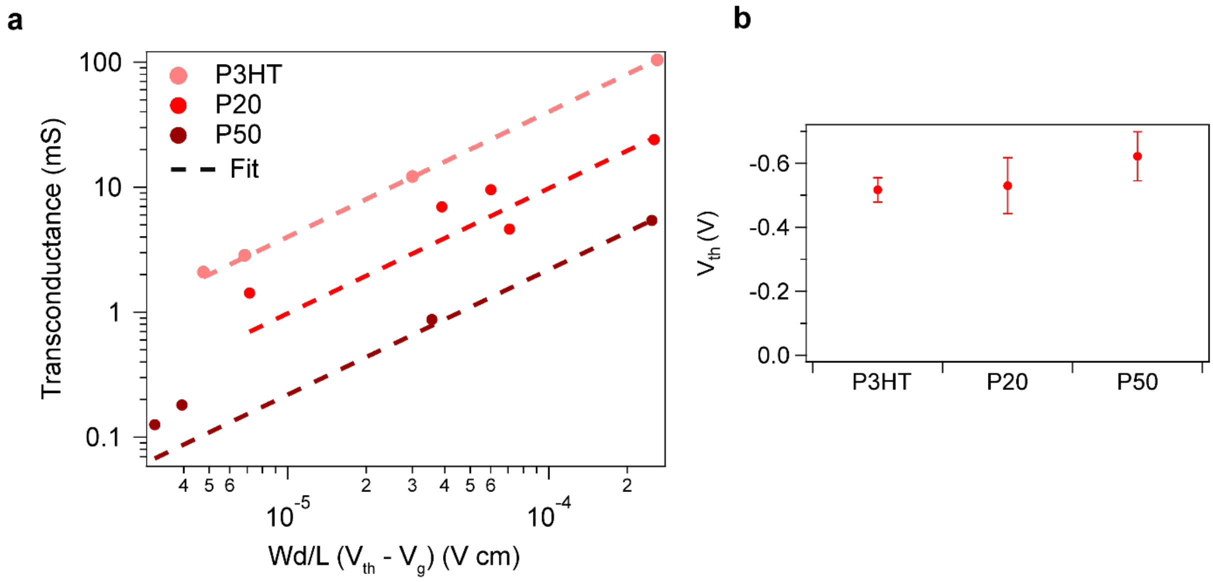

Figure S3: a) Maximum transconductance versus Wd/L (V_{th} - V_g) and b) threshold voltage for P3HT, P20 and P50. Threshold voltages are defined as the x-intercept of the linear fits of the forward $\sqrt{I_{DS}^{Sat}}$ versus V_G, according to $\sqrt{I_{DS}^{Sat}} = \sqrt{\frac{Wd\mu C*}{2L}}\ (V_{th} - V_g)$. The values shown in b) were determined for 4- 5 OECTs for each material and averaged. The errors represent the standard deviation.

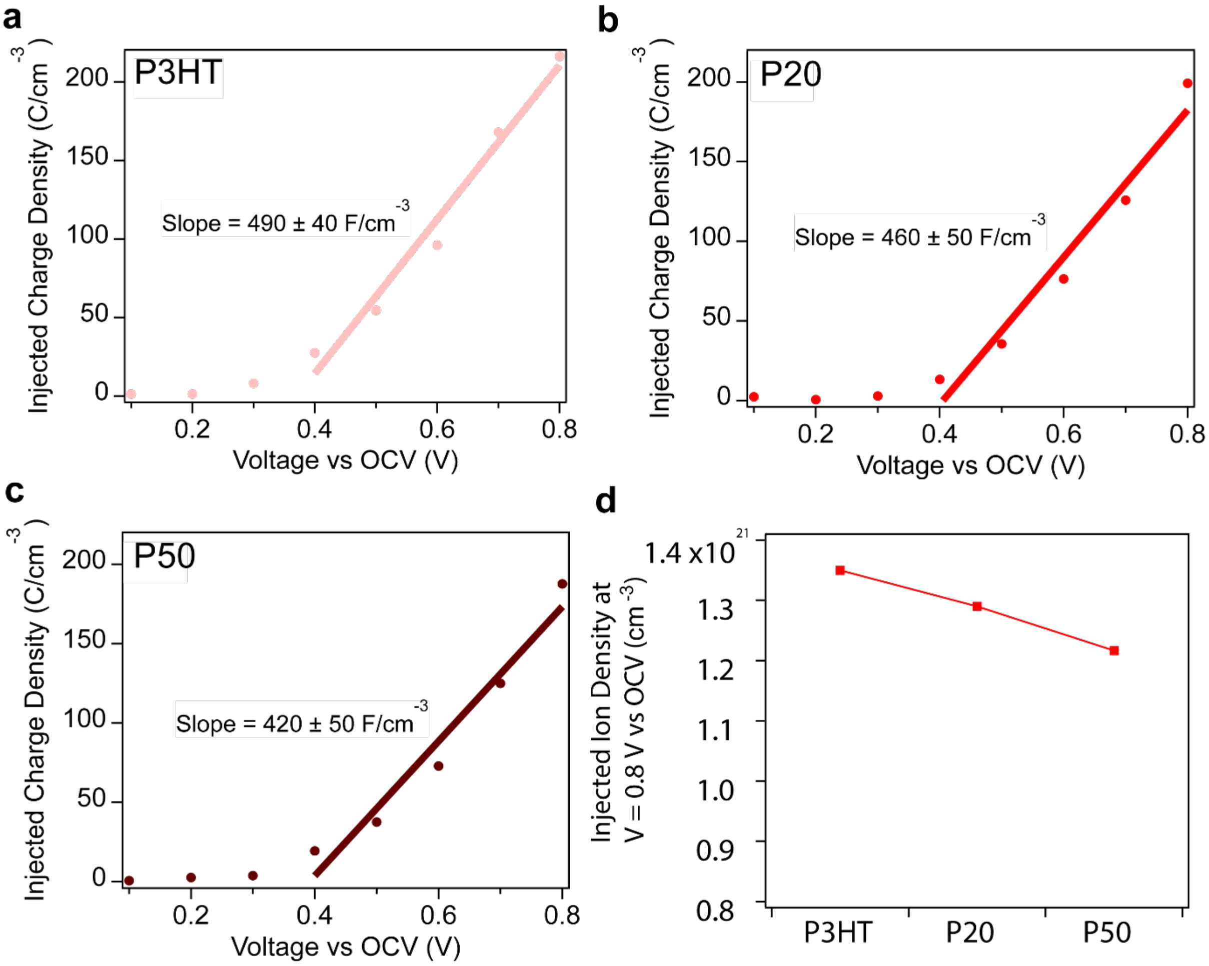

Figure S4: a) Injected charge density obtained during E-QCMD by integrating the current in chronoamperometry measurements with a) poly(3-hexylthiophene) (P3HT), b) P20 and c) P50, as described in detail in previous works.[9] Briefly, we apply 120 s square voltage pulses ranging from 0.1 V to 0.8 V vs OCV. The measured current is integrated, resulting in the injected charge evolution in time. To remove any contributions from faradaic processes, we linearly fit the last 20 s of the injected charge and consider the intercept as the total injected charge for each voltage step. Next, we plot the injected charge density of each film versus the applied voltage, as shown in a, b and c. The slope of the curves is the volumetric capacitance. This method has been shown to give equivalent values to the ones obtained via electrochemical impedance spectroscopy.[10] d) Using the same method, we calculate the density of injected ions at V = 0.8 V vs OCV from the E-QCMD measurements, assuming that one injected hole is equivalent to one injected ion. This assumption is typically made in polymer films E-QCMD studies.[11]

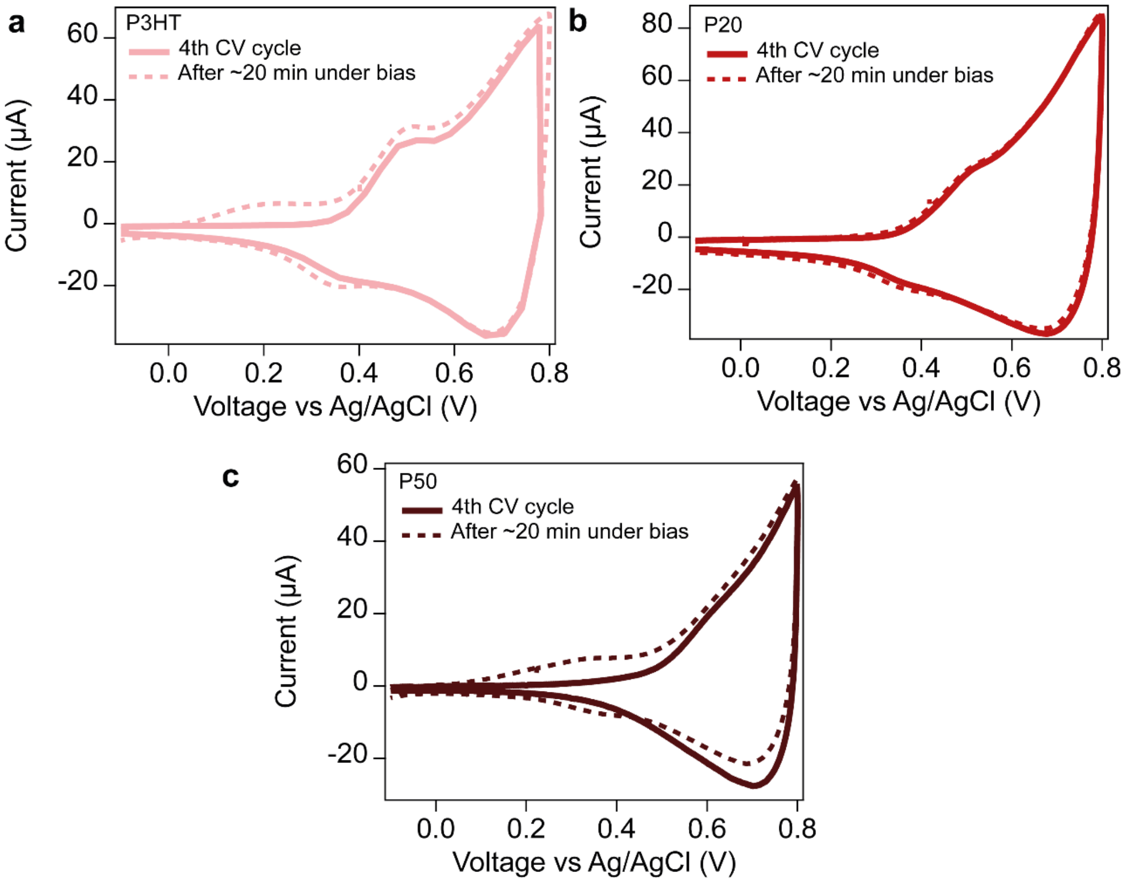

Figure S5: Cyclic voltammetry of a) P3HT, b) P20 and c) P50 thin films in 0.1 M KPF_6 electrolyte with scan rate of 50 mV/s. The solid lines represent voltammograms measured after 3 initial pre-conditioning scans. The dashed lines represent voltammograms measured after running several CV scans at different rates and applying doping/dedoping voltage steps between -0.4 and +0.8 V for about 20 minutes.

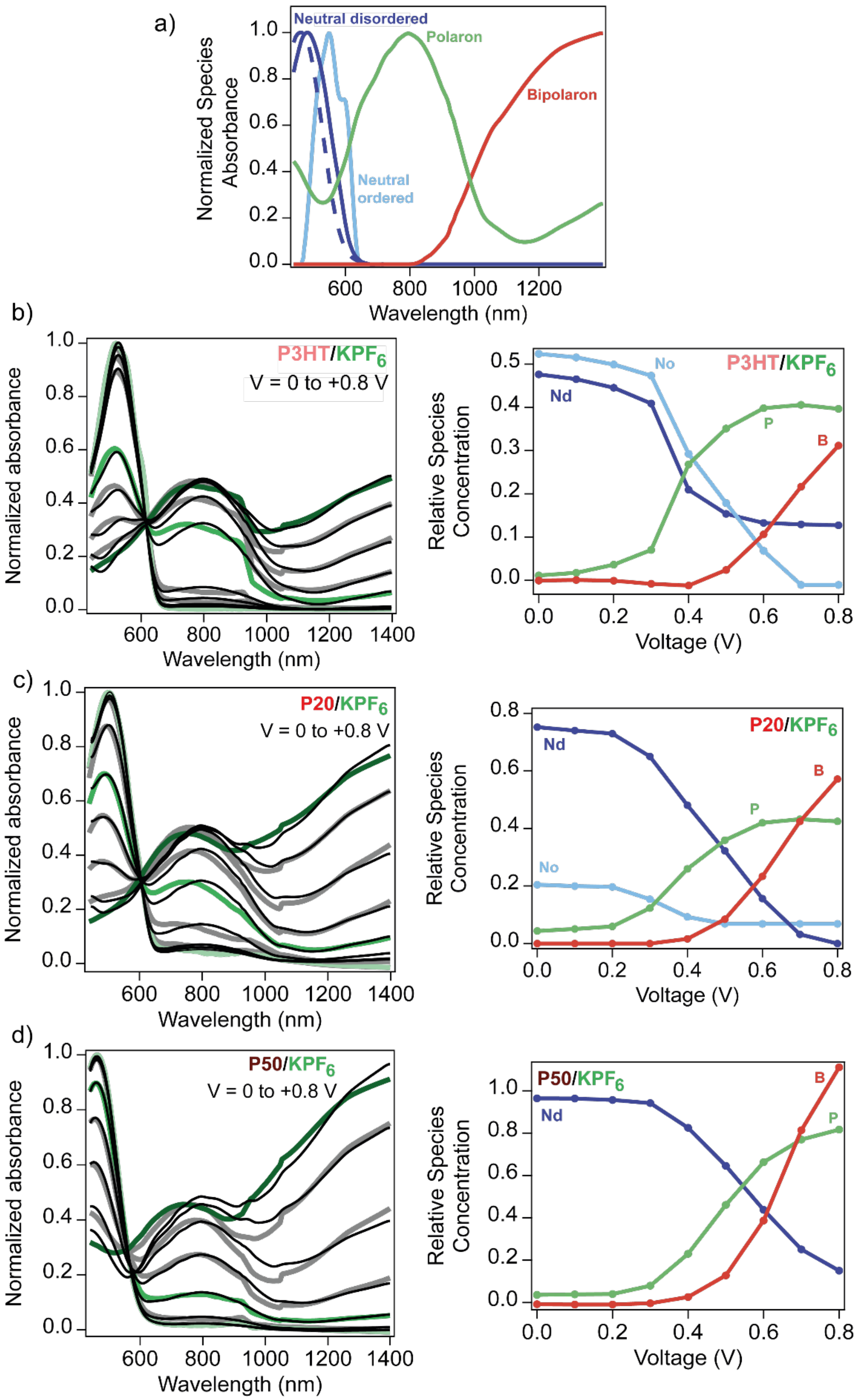

Figure S6: Multivariate Curve Resolution (MCR) fitting of the spectroelectrochemical data related to the films doped in KPF_6. a) Spectral signatures related to neutral disordered (dark blue; solid lines for P20 and P3HT, dashed for P50), neutral ordered (light blue), polaron (green) and bipolaron (red). b), c) and d) show the measured spectra at different voltages in grey and green, and the corresponding MCR fits in black for P3HT, P20 and P50, respectively.

On the right, the relative MCR concentrations of each species are shown. For each polymer film, concentrations were normalized to the total initial concentration of all species. A possible reason for the deviations between the fitted and measured spectra is that the same spectral signatures were used to fit to all films, except for the neutral disordered used in P50. As a result, any spectral shifts that may occur due to differences in film order are not captured by the MCR fitting. Furthermore, the steep decay observed in the polaron band around 950 nm for the P3HT film is likely an artifact caused by the experimental setup.

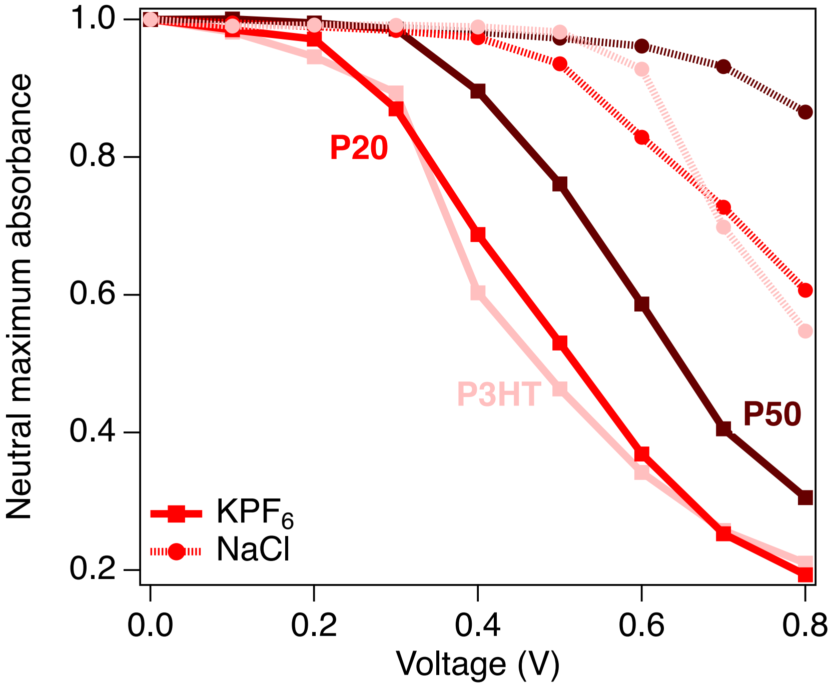

Figure S7: Voltage evolution of the neutral absorbance at the band maximum for the dry P3HT, P20 and P50 films. Solid lines were measured in KPF_6 and dashed in NaCl aqueous electrolyte.

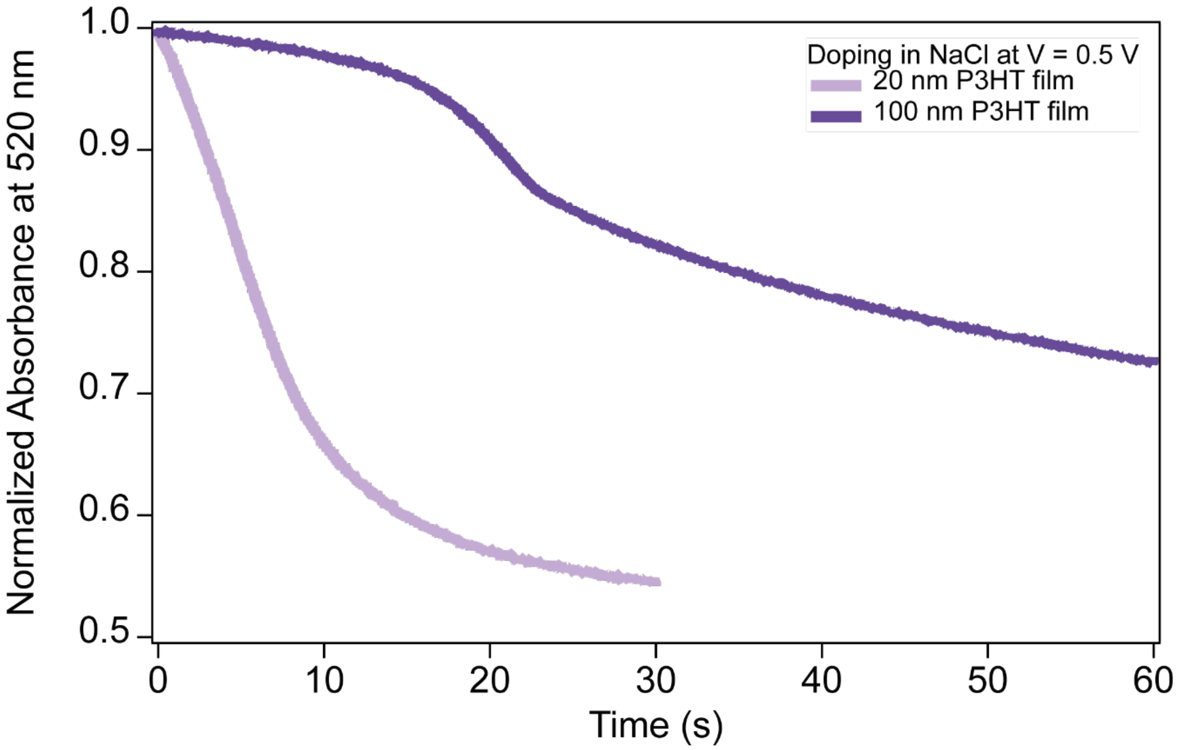

Figure S8: Time-resolved normalized absorbance changes at 520 nm while doping a 20 and a 100 nm P3HT film at V = 0.5 V. The curves were normalized by dividing them by their maximum.

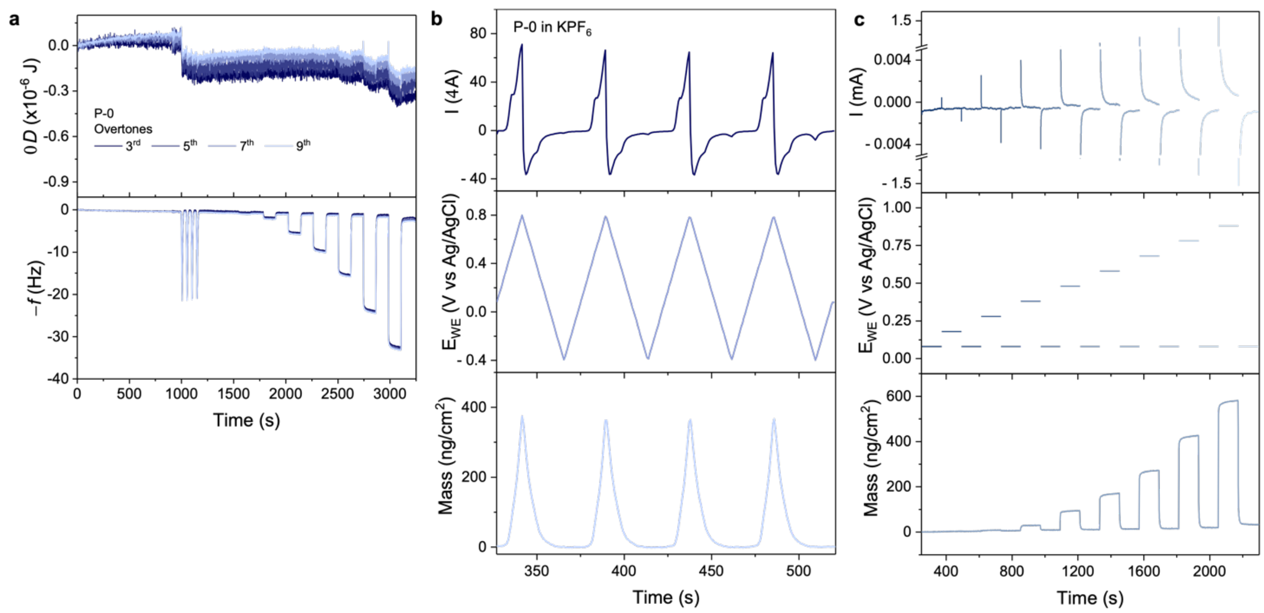

Figure S9: Overview of P3HT E-QCMD measurements: a) dissipation and frequency, current, voltage and mass for the b) CV cycles, and c) chronoamperometry with voltage steps from +0.1 V to +0.8 V versus OCV.

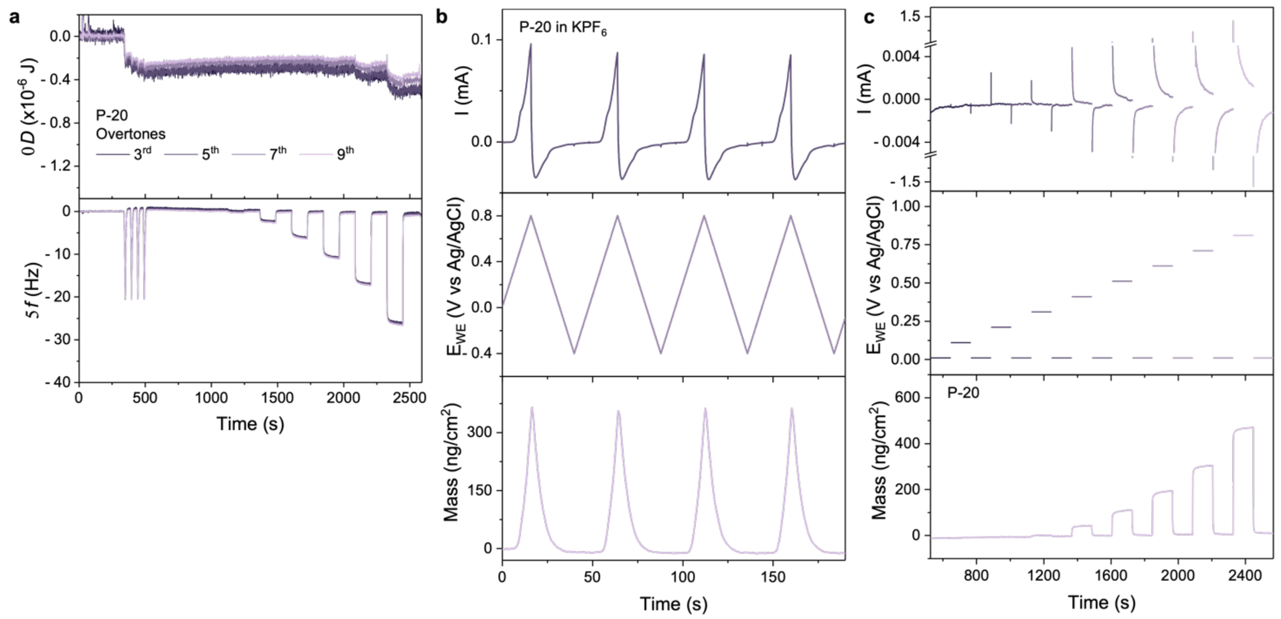

Figure S10: Overview of P20 E-QCMD measurements: a) dissipation and frequency, current, voltage and mass for the b) CV cycles, and c) chronoamperometry with voltage steps from +0.1 V to +0.8 V versus OCV.

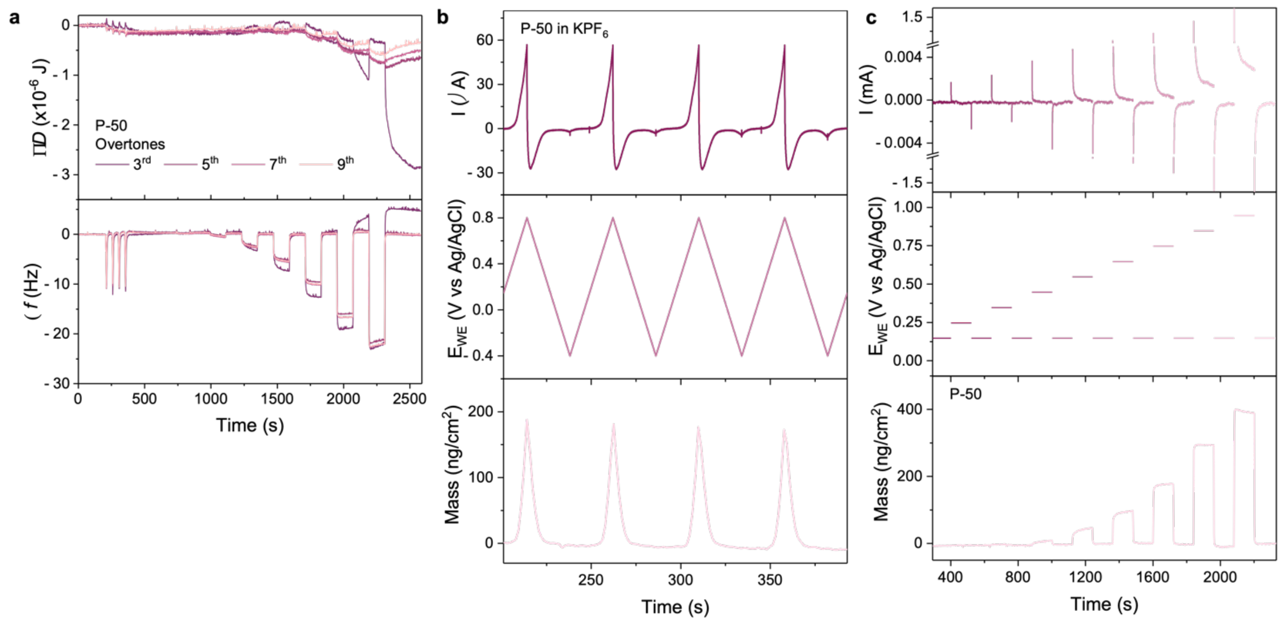

Figure S11: Overview of P50 E-QCMD measurements: a) dissipation and frequency, current, voltage and mass for the b) CV cycles, and c) chronoamperometry with voltage steps from +0.1 V to +0.8 V versus OCV.

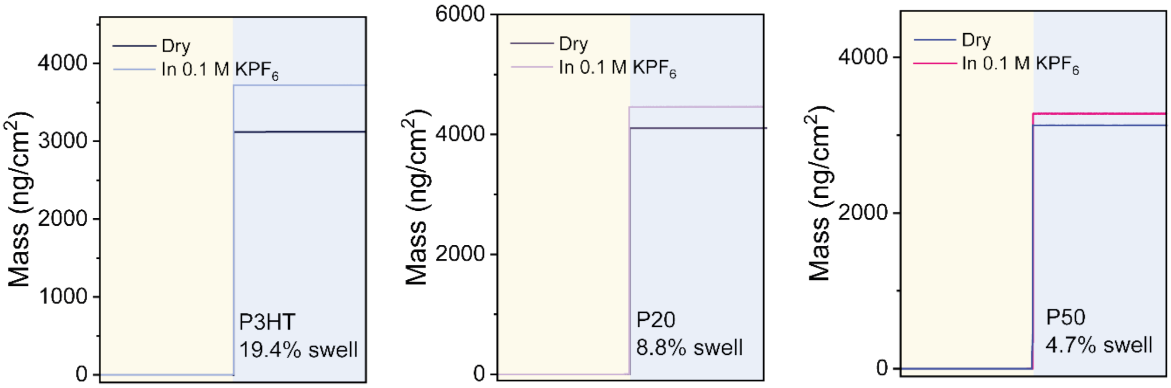

Figure S12: Passive swelling of P3HT (left), P20 (center) and P50 (right) in 0.1 M aqueous KPF_6 electrolyte.

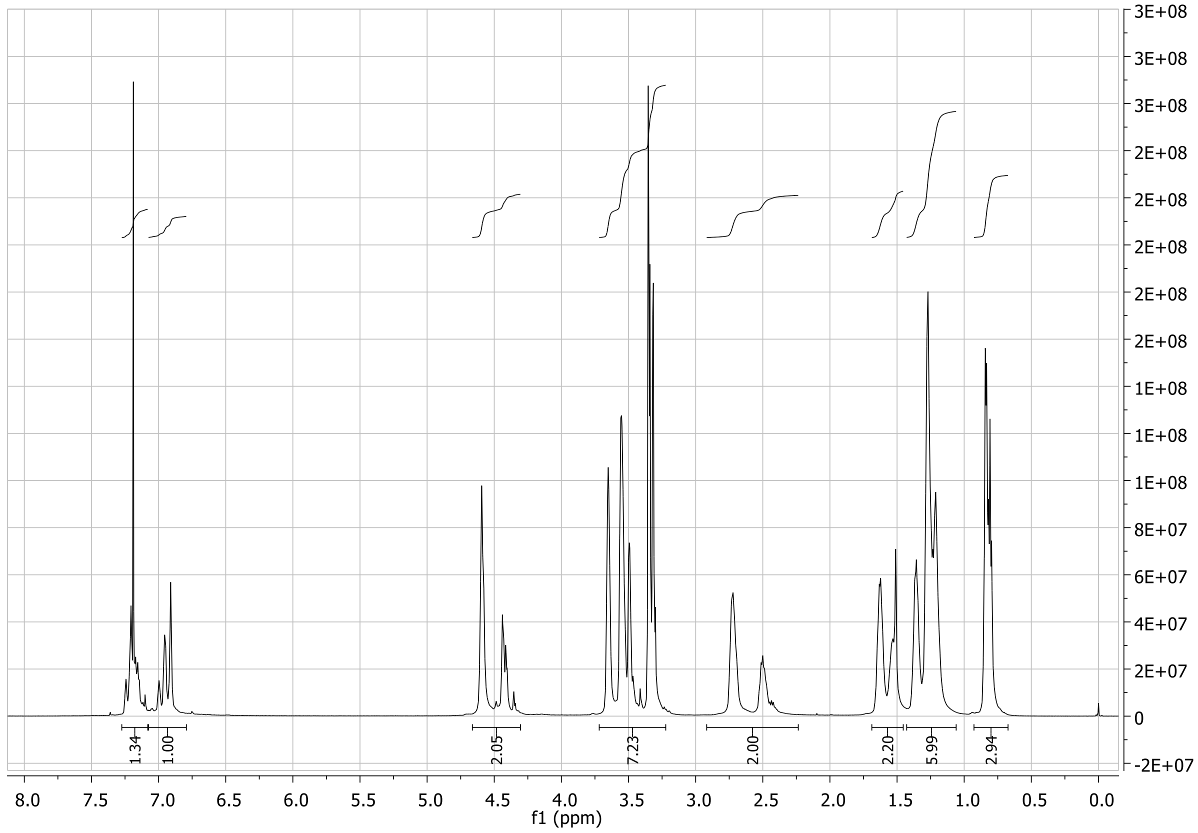

Fig S13. ^{1}H NMR spectrum of P50 recorded in $CDCl_3$ on a 400 MHz spectrometer.

References

[1] L. Q. Flagg, C. G. Bischak, J. W. Onorato, R. B. Rashid, C. K. Luscombe, D. S. Ginger, *J. Am. Chem. Soc.* **2019**, 141, 4345.

[2] L. Q. Flagg, W. Cho, J. Woodcock, R. Li, H. W. Ro, D. M. Delongchamp, L. J. Richter, *Chem. Mater.* **2024**, 36, 1352.

[3] J. Tropp, D. Meli, R. Wu, B. Xu, S. B. Hunt, J. D. Azoulay, B. D. Paulsen, J. Rivnay, *ACS Materials Letters* **2023**, 5, 1367.

[4] P. Schmode, A. Savva, R. Kahl, D. Ohayon, F. Meichsner, O. Dolynchuk, T. Thurn-Albrecht, S. Inal, M. Thelakkat, *ACS Appl. Mater. Interfaces* **2020**, 12, 13029.

[5] S. E. Chen, L. Q. Flagg, J. W. Onorato, L. J. Richter, J. Guo, C. K. Luscombe, D. S. Ginger, *Journal of Materials Chemistry A* **2022**, 10, 10738.

[6] M. Moser, T. C. Hidalgo, J. Surgailis, J. Gladisch, S. Ghosh, R. Sheelamanthula, Q. Thiburce, A. Giovannitti, A. Salleo, N. Gasparini, A. Wadsworth, I. Zozoulenko, M. Berggren, E. Stavrinidou, S. Inal, I. McCulloch, *Adv. Mater.* **2020**, 32, 2002748.

[7] B. Ding, V. Le, H. Yu, G. Wu, A. V. Marsh, E. Gutiérrez-Fernández, N. Ramos, M. Rimmele, J. Martín, J. Nelson, A. F. Paterson, M. Heeney, *Adv. Electron. Mater.* **2024**, 10, 2300580.

[8] M. Moser, L. R. Savagian, A. Savva, M. Matta, J. F. Ponder, Jr., T. C. Hidalgo, D. Ohayon, R. Hallani, M. Reisjalali, A. Troisi, A. Wadsworth, J. R. Reynolds, S. Inal, I. McCulloch, *Chem. Mater.* **2020**, 32, 6618.

[9] a)P. Cavassin, I. Holzer, D. Tsokkou, O. Bardagot, J. Réhault, N. Banerji, *Adv. Mater.* **2023**, 35, 2300308; b)D. Tsokkou, P. Cavassin, G. Rebetez, N. Banerji, *Mater. Horiz.* **2022**, 9, 482.

[10] J. Rivnay, P. Leleux, M. Ferro, M. Sessolo, A. Williamson, D. A. Koutsouras, D. Khodagholy, M. Ramuz, X. Strakosas, R. M. Owens, C. Benar, J.-M. Badier, C. Bernard, G. G. Malliaras, *Sci. Adv.* **2015**, 1, e1400251.

[11] A. D. Easley, T. Ma, C. I. Eneh, J. Yun, R. M. Thakur, J. L. Lutkenhaus, *Journal of Polymer Science* **2022**, 60, 1090.
